# Neurogenic Bowel Dysfunction in Patients with Spinal Cord Injury and Multiple Sclerosis—An Updated and Simplified Treatment Algorithm

**DOI:** 10.3390/jcm12226971

**Published:** 2023-11-07

**Authors:** Fredrika S. Magnuson, Peter Christensen, Andrei Krassioukov, Gianna Rodriguez, Anton Emmanuel, Steven Kirshblum, Klaus Krogh

**Affiliations:** 1 Department of Hepatology and Gastroenterology, Aarhus University Hospital, 8200 Aarhus, Denmark; 2 Department of Surgery, Aarhus University Hospital, 8200 Aarhus, Denmark; 3 International Collaboration of Repair Discoveries (ICORD), Department of Medicine, Division of Physical Medicine and Rehabilitation, The University of British Columbia, Vancouver, BC V5Z 1M9, Canada; 4 Physical Medicine and Rehabilitation, Spinal Cord Injury Medicine, University of Michigan Health, Ann Arbor, MI 48108, USA; 5 GI Physiology Unit, University College London Hospital, London WC1E 6DB, UK; 6 Kessler Institute for Rehabilitation, West Orange, NJ 07052, USA; skirshblum@selectmedical.com; 7 Department of Physical Medicine and Rehabilitation, Rutgers New Jersey Medical School, Newark, NJ 07103, USA

**Keywords:** neurogenic bowel dysfunction, multiple sclerosis, spinal cord injury, treatment algorithm, treatment pyramid

## Abstract

Neurogenic bowel dysfunction (NBD) is a common condition in individuals with spinal cord injury (SCI) or multiple sclerosis (MS). It usually entails constipation, difficult evacuation of the rectum, and fecal incontinence (FI); often in combination. It is highly burdensome for affected patients and is correlated with poor quality of life. The current treatment algorithm, or treatment pyramid, does not completely correspond to actual clinical practice, and the known and classical pyramid contains both treatments still in their experimental stage as well as several treatments which are not available at all treatment centers. Thus, an updated treatment algorithm is called upon, and the authors of this paper therefore propose a simplified version of the treatment pyramid, aiming to guide clinicians in treating NBD.

## 1. Introduction

Neurogenic bowel dysfunction (NBD) is a common complication in persons with spinal cord injury/disorder (SCI/D) and people with multiple sclerosis (MS). Up to 80% of persons living with sequelae of SCI/D and approximately two thirds of those with MS have NBD [1,2,3,4,5]. NBD ranks among the three most severe consequences in persons with SCI/D [6,7]. The typical presentation of NBD is a combination of constipation, with impaired rectal evacuation and significant time spent on bowel care, and fecal incontinence (FI). Understandably, NBD often results in a severe restriction of social activities and negatively affects quality of life [3,5,8,9,10,11,12]. The underlying pathology includes slow transit of stool through the colon, poor rectal evacuation from the pelvic floor, rectal sphincter muscle impairment (weakness and/or dyssynergia), reduced or absent anorectal sensation, and loss of voluntary anal sphincter control.

The pathophysiology of NBD is complex and multifactorial; hence, symptoms can vary. Thus, a meticulous outlining of each individual’s bowel pattern is of essence in order to provide tailorized and optimized care [2,13,14]. In individuals with SCI, both the severity (complete/incomplete) and the neurological level of spinal cord injury is pivotal. The severity of neurological damage is often classified by the American Spinal Injury Association (ASIA) Impairment Scale and often described in combination with the International Standards for Neurological Classification to supply the level of spinal injury based on neurological examination. A complete injury would correspond to ASIA A (no motor or sensory function preserved below the neurological level) and ASIA B (no motor function but preserved sensory function). ASIA C and D represent motor incomplete spinal injury, and E is normal function preserved [15,16,17,18]. However, it has been questioned how well the ASIA Impairment Scale can anticipate the magnitude of bowel issues [19]. Moreover, some previous studies have conjectured that the severity of NBD is most profound in patients with a complete injury but concluded that symptoms are still common in patients with an incomplete injury, albeit possibly more fluctuating [15,19].

In addition to evaluating neurological damage, it is furthermore recommended to assess remaining autonomic function when determining bowel patterns. As recommended by the ASIA Autonomic Standard Committee, autonomic assessment should include the evaluation of sacral reflexes, sphincter tonus and contraction, bowel method, awareness of bowel fullness/need for bowel emptying, and ability to prevent bowel incontinence [20,21].

Bowel dysfunction subsequent to neurological damage is usually divided into reflexive bowel or areflexive bowel, dependent on whether the injury lies above or within/below the conus medullaris [16,19]. Some guidelines, as well as authors, suggest that reflexive/areflexive bowel might call for different treatment approaches [2,14,22]. Patients with reflexive bowel will often have a slow colonic transit time, hyperreflexic/hypertonic rectal wall and external anal sphincter (EAS), and loss of voluntary control of EAS [22,23]. The result is retained stool/constipation and FI, the latter due to defecation occurring solely by reflexes when the rectum is distended by the retained stool [16,19]. Patients with areflexive bowel will likely have lost centrally mediated peristalsis and defecation reflexes and concomitantly have reduced anorectal sensation and tone of EAS. This potentially results in constipation as well as concomitant and frequent FI due to impaired reflexes, reduced anal sensation, and laxity of EAS [16,19,22].

Adding to the perplexity of NBD, disturbances of the parasympathetic and/or the enteric nerve system due to neurological damage may further contribute to gastrointestinal dysmotility in NBD. It is believed to cause perturbations in ion secretion and release several neurotransmitters in the intestinal wall, affecting contractions of the smooth muscle layer, as well as the interstitial cells of Cajal, resulting in dysmotility [24]. Ion secretion is also considered to have substantial effect on the absorption of electroneutral NaCl, an important role of the small intestine and large bowel. However, this has primarily been shown in animal models and discussed in relation to inflammatory and infectious diseases and not NBD. Nonetheless, dysregulated water absorption in the gastrointestinal tract may further exacerbate bowel symptoms [25].

The complexity and variety of NBD, as demonstrated above, conduce to the challenges that can arise in choosing bowel treatment. As previously mentioned, some of the existing guidelines regarding NBD, e.g., Multidisciplinary Association of Spinal Cord Injured Professionals (MASCIP) guidelines for NBD in individuals with SCI and other central neurological conditions [14] and Clinical Practice Guidelines (CPGs) for NBD in adults after SCI [2], suggest different approaches based on the diagnosis of reflexive or areflexive bowel. This adds to the complexity of which treatments to initiate, when, and for whom, and emphasizes the need for a clear and simple approach when designing treatment for bowel care.

Regarding MS patients, pathophysiology is likewise complex. It is related to lesions of the central nervous system and resembles that of SCI patients. However, due to the nature of the disease and in contrast to SCI, lesions often vary in both severity and location over the course of time. Consequently, so will symptoms, albeit often in a progressive manner [3,16,19].

Considering the variations in the etiology, pathophysiology, and symptoms of NBD, effective treatment presents a challenge and individual evaluation is imperative [2,26].

The challenge of NBD forms an ongoing focus of research and clinical investigation. Unfortunately, little structural research has been conducted [5,11,19,27]. Despite almost three decades of increasing attention to NBD and the introduction of new treatment modalities, most patients with NBD have been using the same method for bowel care for years, even if it is not effective. Treatment of NBD today aims to improve transit through the colon, enhance rectal evacuation, if abnormal, and restore control over time and place of defecation. Treatment should be tailored to the individual and a stepwise approach has been endorsed, commonly illustrated by the so-called “treatment pyramid” [28]. Basic treatment includes diet, physical activity, and oral laxatives, not seldom in combination with added assisted rectal evacuation such as digital anorectal stimulation or evacuation, suppositories, and/or mini enema. If such measures are insufficient, most clinicians recommend transanal irrigation (TAI) [2]. Surgical intervention is reserved for those not responding sufficiently well to conservative treatment [19].

**Figure 1:**
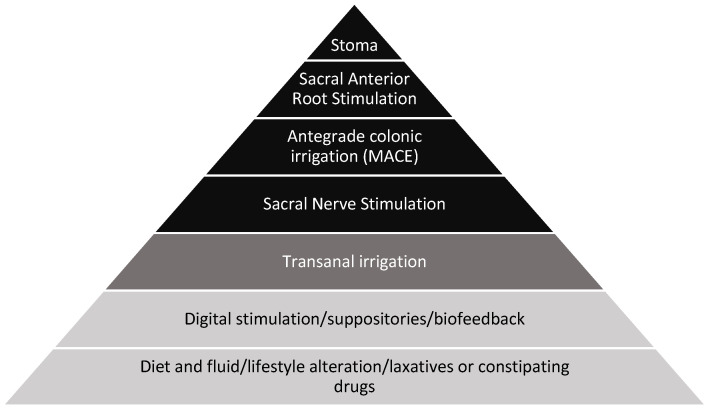
Existing treatment pyramid presented in “Consensus review of best practice of transanal irrigation in adults” by AV Emmanuel et al. [28].

The current version of the “treatment pyramid” (see Figure 1) does not sufficiently reflect clinical practice, as some treatment modalities included remain experimental while others are only available in very few centers [14]. Hence, a simpler treatment algorithm, reflecting best clinical practice and only including commonly available treatment modalities, is warranted. At the same time, emerging treatments, or those only available at few centers should be described outside the general algorithm as optional or experimental.

**Figure 2:**
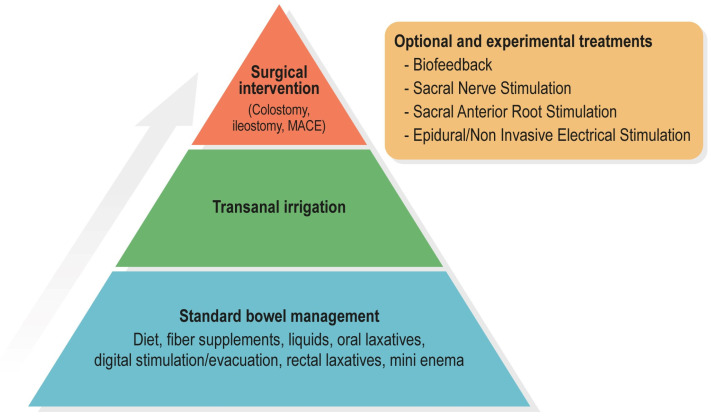
Simplified treatment algorithm for neurogenic bowel dysfunction in persons with spinal cord lesions or multiple sclerosis. Optional and experimental treatments can be considered where available and in the presence of eligible patients.

Thus, the aim of the present paper is to present a new and more practically oriented version of the treatment algorithm or “treatment pyramid” for NBD which is in line with recent guidelines and best evidence available (see Figure 2). The proposed treatment pyramid therefore only contains commonly available steps of NBD treatment, starting with standard bowel management, including oral medications and rectal treatments followed by TAI, with surgery as the last stage. Experimental treatments are presented separately. Since the purpose of this paper is to facilitate treatment of NBD, we have included a brief description of symptom assessment before each step of the pyramid is further elucidated.

We hope this new version of the treatment pyramid will facilitate treatment strategies and help clinicians navigate through the complex and perplexing field of NBD.

## 2. Methods

A literature search was conducted in the PubMed/MeSH database using the terms “neurogenic bowel dysfunction” in altering combinations with “spinal cord injury”, “multiple sclerosis”, “pathophysiology”, “transanal irrigation”, “diet”, “laxatives”, “stoma”, “malone antegrade continence enema”, “sacral nerve stimulation”, “sacral anterior root stimulation”, “digital/mechanical rectal stimulation”, and “quality of life”. Furthermore, the bibliographies of pertinent articles were scrutinized to further identify additional articles of relevance.

## 3. Assessment of NBD and Treatment Evaluation

**Figure 3:**
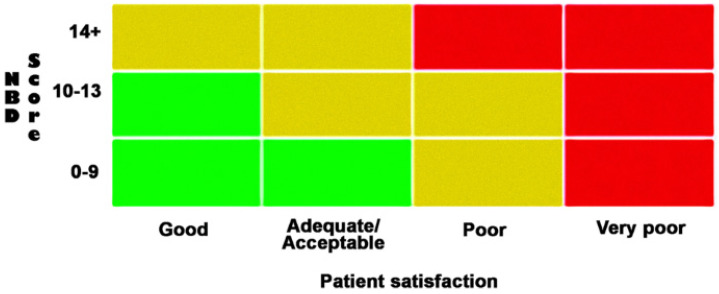
MENTOR tool combined with NBD score. MENTOR tool/picture from article “Creation and validation of a new tool for the monitoring efficacy of neurogenic bowel dysfunction treatment on response: the MENTOR tool”, by Emmanuel et al. [29].

In daily practice, NBD is primarily a clinical diagnosis. Due to the nonuniform nature and causes of NBD, there is no single measure that can be used to assess and manage dysfunctions across all clinical conditions [5,10]. The most commonly used tool to evaluate the severity of NBD as well as the effect of current treatment is the 10-item NBD score, validated in persons with SCI and translated into more than 12 languages [8,29,30]. The NBD score is part of the International Spinal Cord Bowel Function Basic Data Set [30]. The recently developed Monitoring Efficacy of Neurogenic Bowel Treatment On Response (MENTOR) tool incorporates the NBD score, patient satisfaction with bowel management, and special attention symptoms [29]. It is meant as a simple tool to identify persons with NBD in need of a change in strategy for bowel management. Hence, the MENTOR tool classifies NBD into the three categories: Observe (green), Discuss (yellow), and Act (red) (see Figure 3) [29]. The utility of the NBD score and MENTOR tool in patients with MS has been questioned [31].

Evaluation of NBD and treatment efficacy is carried out when commencing treatment, and from hereon when deemed necessary in each individual. As a minimum, MASCIP guidelines suggest a re-evaluation would be deemed appropriate in relation to changes in lifestyle, diet, physical ability, medical conditions, worsening of symptoms, and cease of patient satisfaction with current treatment and with check-ups. Additionally, they recommend more frequent evaluation during the acute phase of the neurological condition and rehabilitation [14]. CPGs further add a recommendation of annual assessment during treatment [2]. Furthermore, assessment includes medical history, including information on bowel function before NBD, well as a physical examination. Some clinicians favor a bowel diary. In selected patients, basic clinical information and examination can be supplemented with image diagnostics (X-ray, computed tomography (CT), magnetic resonance imaging (MRI)), gastrointestinal transit times, and anorectal manometry [2,13,32]. A more comprehensive description of the different modalities and indications of these is beyond the scope of this paper.

## 4. Standard Bowel Management

Standard bowel management (SBM) usually includes advice on diet and fluid intake and timing of defecation. Usually, this is supplemented by oral laxatives or digital anorectal stimulation/evacuation or rectal laxatives, depending on patient preference and the main symptoms.

### 4.1. Diet, Fibers, and Fluids

A personalized treatment program for bowel care is considered a standard of care in patients with SCI/D or MS. Aspects of diet, fluids, physical activity, defecation method, and laxatives are thoroughly considered, with the aim of facilitating and ensuring regular, controlled evacuations of stool and preventing fecal incontinence. It is important to tailor bowel management programs to each patient, as daily intake of food, fluids, and other medications, level of physical activity, extent of neurological damage, as well as bowel evacuation can vary greatly [13,33].

Fiber and fluid intake are substantial influences on bowel movements, not only in the general population, but also among patients with SCI and NBD [2,34]. Unfortunately, scientific evidence from structured studies regarding fiber intake within the SCI population is sparse. Nonetheless, the existing literature seems to support that patients with SCI and concomitant NBD who regulate diet, fiber, and fluid intake have less abnormal frequency of defecation, time spent on bowel care, and need for invasive interventions [2,9,35]. Within the SCI population, transit time might be unaltered or even prolonged by increased fiber intake. However, the definite effect of fibers on colonic function in NBD remains to be determined [2,13]. Furthermore, excessive fiber intake can cause bloating and abdominal distension. The amount of fiber intake is therefore recommended to be gradually introduced and individualized, ensuring tolerability and keeping possible side effects at a minimum. Furthermore, the type of fiber also affects stool consistency [13]. Although formalized studies within the MS population are lacking, the literature encourages the same approach towards MS patients with NBD [2,9,19,26,32]. Concerning general diet, besides the amount of fibers, little research has been carried out within the SCI and MS populations.

Regarding fluid intake, CPGs state that euhydration is likely to be the most appropriate recommendation, although it emphasizes the importance of individualizing the precise amount to optimize stool consistency while at the same time preventing bladder symptoms [2,36].

### 4.2. Timing

Both CPGs and MASCIP guidelines discuss the enhancing effect of the gastrocolic reflex on bowel movement and recommend bowel evacuation in relation to this. Furthermore, it is recommended to plan bowel evacuation regularly to ensure the presence of stool in the rectum and to prevent uncontrolled or ineffective bowel movements [2,13,14].

### 4.3. Oral Laxatives

Despite regulating diet, fluids, and physical activity, the additional use of oral laxatives to facilitate and regulate bowel movements is often required. Just like the rest of bowel management, the choice of laxative and timing of initiation should be based on patients’ individual challenges and introduced in a stepwise approach in patients with both SCI and MS. Dividing oral laxatives according to their mechanism of action, oral laxatives can be classified as bulking/osmotic, stimulants, prokinetics, and secretory drugs.

#### 4.3.1. Bulking Laxatives

Bulking laxatives consist primarily of fibers and fiber supplements. However, clinicians need to be aware of the type and amount of fiber for optimal benefits and minimizing side effects [37]. There are both soluble and insoluble fibers, as well as fibers of varying sizes. Consequently, their effects on the bowel differ. Soluble fiber, like psyllium, can hold a large amount of water, preventing the desiccation of stool and simultaneously loose stools. Large, insoluble fiber, e.g., wheat bran, exerts mechanical irritation on the mucosa, stimulating secretion and resulting in a moister, easier-to-pass stool. On the contrary, smaller insoluble fibers are not able to stimulate secretion, merely drying out stool and contributing to a constipating effect [37].

#### 4.3.2. Osmotic Laxatives

Osmotic laxatives have the purpose of softening stool. Through increasing moisture and water content in stool and in the intestinal lumen, easier-to-pass stool is achieved. Furthermore, a presumed additional effect is the indirect stimulation of peristalsis due to increased mass within the lumen of the gut [2]. Examples of osmotic laxatives are (international nonproprietary name, INN) docusate sodium, docusate calcium, PEG (polyethylene glycol), magnesium hydroxide, and lactulose. These are generally available, mostly inexpensive, and have mild adverse events. Hence, they should be the first type of laxatives introduced [2,16].

#### 4.3.3. Stimulant Laxatives

Stimulant laxatives, e.g., bisacodyl and sennosides, stimulate motility and secretion of water and electrolytes into the colon and possibly inhibit water absorption as well [2,16]. These can either supplement or replace osmotic laxatives.

#### 4.3.4. Prokinetics

Prokinetics enhance bowel peristalsis. Prucalopride, a serotonin HT4 receptor agonist, is the most frequently used among prokinetics. A small pilot study demonstrates positive results of prucalopride in patients with NBD due to SCI; however, precise use is yet to be established [38]. Other prokinetic drugs, including neostigmine and metoclopramide, have significant side effects, and therefore are not considered a standard part of NBD treatment but rather for use in extreme cases or for bowel emptying before procedures in patients with SCI [16].

Secretory drugs such as linaclotide reduce visceral pain and stimulate peristalsis by increasing intestinal secretion, but their use remains to be validated within the NBD population [16]. In patients with suspected additional opioid-induced bowel dysfunction, opioid antagonists such as naloxegol can be indicated [39].

A recent systematic review of pharmacological treatment within NBD found little methodological research on the topic. Based on studies in other populations and expert opinions, the authors recommend osmotic agents to be the first intervention before stimulants and prokinetics, while still emphasizing the importance of individual assessment [16]. Another review likewise found sparse structural scientific studies regarding pharmacological treatment within the NBD community, calling for more [27].

### 4.4. Digital Anorectal Stimulation/Evacuation

Many patients with NBD make use of mechanical anorectal stimulation, either instead of or in combination with laxatives. Its mechanism of action is to stimulate anorectal reflexes, thereby enhancing evacuation. Mechanical anorectal stimulation primarily consists of digital rectal stimulation (DRS) and chemical stimulation with a rectal laxative. According to CPGs for NBD treatment in adults with SCI [2], between 15% and 72% of the SCI population use this technique. These patients report fewer episodes of unintentional bowel emptying and subsequently higher quality of life, as compared to patients using oral and rectal laxatives exclusively. However, DRS could potentially be associated with some complications, including anal fissures, hemorrhoids, and autonomic dysreflexia (AD) [2]. Two recent reviews regarding DRS seem to ascertain the fact that digital rectal evacuation is in fact effective, simple, and inexpensive [40,41]. However, they both call upon further structured interventional studies, as current reports are primarily based on observational short-term studies, while very few have isolated the sole effects of DRS, its long-term effects, and risks of complications [40,41]. DRS is most commonly used by individuals with reflexive bowel [2]. Much less is known about DRS in patients with MS.

### 4.5. Rectal Laxatives

Rectal laxatives are also considered a major part of the management of NBD. They consist of suppositories, mini enemas, and enemas. They can be used alone or in combination with oral laxatives and/or as chemical rectal stimulation. Rectal medications have the benefit of giving the patient control over the time and place of evacuation, hence helping prevent fecal incontinence as well as enhancing expulsion of stool [2,16].

#### 4.5.1. Rectal Suppositories

Rectal suppositories are semi-solid rectal medications to be inserted through the anal canal. Bisacodyl and glycerin are active substances commonly used in rectal suppositories. Glycerin is a mild irritant and lubricant which stimulates rectal contractions and softens stool. Bisacodyl also possesses the above-mentioned mechanisms of action in the rectum. Bisacodyl suppositories can be PEG-based or vegetable oil-based, and several studies have found PEG-based bisacodyl suppositories to be superior in facilitating bowel evacuation [42,43]. Glycerin suppositories are less of a chemical irritant than bisacodyl; however, they still have a beneficial effect on transit time in the right colon and softening stool. They are easy to manage and are still commonly used [2,13,16,44].

#### 4.5.2. Mini Enema

A mini enema is an alternative to suppositories. Its primary active substances are docusate sodium combined with glycerol and PEG, with or without benzocaine [2]. Studies have shown that the effect of PEG-based bisacodyl suppositories is comparable with that of docusate sodium–benzocaine mini enemas. A possible inconvenience of the mini enema is that it is slightly more difficult to manage than suppositories. On the other hand, a possible superiority of the mini enema is that some authors find shorter evacuation times with docusate sodium mini enemas vs. bisacodyl suppositories. However, the base type of the latter has not been specified in these studies, leaving results as to which bisacodyl suppositories are referred to open for interpretations [16,44,45]. A low-volume tap water enema can also be administered with commercially available systems like Qufora IrriSedo MiniGo™ (Qufora, Allerød, Denmark) or Navina Mini™ (Wellspect, Molndal, Sweden).

## 5. Transanal Irrigation

Transanal irrigation (TAI) is a retrograde colonic flush-out by water introduced via the anal canal. Usually, the irrigation fluid is tap-water at approximately 36–38 degrees Celsius [2,28]. Several devices have been designed to facilitate TAI, in which the different utensils usually consist of either a rectal cone or a balloon catheter for insertion into, and sealing of, the anal canal during the inflow of water. This is connected to either a pump or a gravity-based reservoir system for introducing the water [28]. Examples are different versions of, e.g., Peristeen™ by Coloplast (Humlebaek, Denmark), Navina Irrigations systems ^TM^ by Wellspect (Molndal, Sweden), Qufora IrriSedo Flow™ by Qufora (Allerød, Denmark) or Aquaflush Lite™ by Renew Medical (London, UK). The pump can either be gravity-based or manually or electrically operated. The choice of device will depend on the patient’s condition, mobility, dexterity, and preference [46].

Furthermore, TAI can be divided into low-volume or high-volume TAI. The use of low-volume devices (administering up to 250 mL) will empty the rectum only and is hence appropriate in assisted rectal evacuation [47]. In high-volume TAI, amounts will usually lay somewhere between 250 and 1500 mL, most commonly approximately 700 mL [46,48]. Usually, high-volume TAI will empty the left colon from the splenic flexure to the rectum [49].

TAI’s mechanisms of action are considered to be stimulated colonic peristalsis due to mechanical pressure on the colonic wall exerted by the incoming water, and/or a simultaneous mechanical wash-out facilitated by the water [4].

A randomized controlled trial performed by Christensen et al. in 2006 found that in patients with NBD due to SCI, TAI was significantly more effective than a standard bowel management program, considering parameters such as constipation, FI, and quality of life [1]. However, TAI is usually recommended to NBD patients for whom a conservative regime has been inadequate [2,29,50]. Hence, it is proposed as the next level of intervention, after SBM and assisted rectal evacuation. Previous studies have explored predictors of successful outcomes of TAI. Factors such as NBD (as opposed to idiopathic constipation or rectal surgery), the male sex, constipation with concomitant FI, and prolonged colonic transit time were all predictors of a positive outcome [50]. However, other studies failed to find common traits to predict a positive or negative outcome and consistent predictors of a positive outcome of TAI remain uncertain [51]. Most authors, moreover, underline that patient motivation and psyche are important [28]. TAI is, however, an established and viable treatment for constipation, extensive time spent on bowel emptying, and FI. Furthermore, it is known to improve quality of life as well as having the same, or higher, level of patient satisfaction as SBM [1,2,11,50,52]. TAI remains the most evidence-based treatment of NBD in patients with SCI.

Within the MS population, several studies concluded that TAI improved bowel symptoms and quality of life in persons with NBD [5,53,54]. Amongst MS patients, one study found that the sole predictive factor for successful treatment was impaired anal electrosensitivity at baseline [5]. This would be in line with tendencies previously seen in an earlier study, where patients with more severe incontinence symptoms, better tolerability to rectal catheters, as well as a better awareness of their own health had the best outcome with TAI [54].

## 6. Surgical Interventions

### 6.1. Stoma

A colostomy or ileostomy is usually reserved for patients who fail to respond to all conservative measures. The surgical procedure, albeit considered a routine procedure, is not without risk of complications, such as stoma prolapse, stoma-related hernia, strictures, skin complications, and bowel obstruction. Rectal discharge may also still occur, even in the presence of a stoma [2]. Several studies have investigated the results of stoma formation in the SCI population and concluded that stoma in general is well tolerated and accepted, increases independence and decreases time spent on bowel management, as well as improves quality of life [55,56,57,58]. Most patients in these studies had received a colostomy; however, some had an ileostomy and a few ended up with both eventually, primarily due to complications. Preferences of ileostomy vs. colostomy are not particularly discussed nor compared in these studies [55,56,57,58,59]. The decision on stoma formation should be well considered. A shared decision between the patient and surgeon is pivotal, and patients ought to be well informed on both risks of complications as well as positive gains. There are no official guidelines on when a stoma operation should be performed. Thus, indications should be sought and contemplated individually [2].

### 6.2. The Malone Antegrade Continence Enema (MACE)

MACE is a procedure where a stoma is shaped from the appendix, creating a channel between the skin and bowel through which a catheter can be inserted and antegrade irrigation can be performed. If the appendix is not available or suitable for creating an appendicostomy, a coecostomy catheter (Chait catheter) can be placed, either guided by colonoscopy or with a CT scanner [60,61]. The procedure is reversible [62]. Common complications are infections, stenosis of the canal, leakage, and bowel obstruction. Complications are reported in various amounts. Nonetheless, the results of the MACE system are good and several studies have found beneficial outcomes in a large portion of patients—up to 83% in some studies, both regarding bowel management and quality of life [2,63,64]. The greatest issue with MACE would probably be availability, as it is not performed at every clinical center.

In MS patients suffering from severe NBD for whom a conservative regimen has been inadequate, stoma formation also shows positive results regarding bowel management and quality of life [26,65].

## 7. Experimental Treatments

There are treatments for NBD which are still in their infancy or are not widely accessible, and therefore not part of clinical standard practice.

### 7.1. Biofeedback (BF)

BF is a technique to help regulate and train pelvic floor muscles, relearn how to interpret signals from the colon and the rectum, and generate an appropriate muscular response. A device designed to facilitate exercises of the external anal sphincter is used and visualization feedback is given to the patient regarding how to adapt and optimize pelvic floor muscle training [26]. It can help some patients gain control over the urge to defecate and increase strength and ability to coordinate sphincter contraction and relaxation. BF is suggested by some authors as the next level of treatment when regulation of diet and laxative/constipation medication fails [26,65]. These authors also state that BF can be a beneficial treatment for SCI patients with an incomplete injury [26,66].

### 7.2. Sacral Nerve Stimulation (SNS) and Sacral Anterior Root Stimulation (SARS)

SNS and SARS involve surgery and placing electrodes usually stimulating the afferent branches of S3 and the anterior roots of S2–S4, respectively [2,67]. Both modalities have demonstrated good results, improving constipation, frequency of defecation, and FI. SNS is considered a minor surgery with limited complications, although it has primarily been investigated in SCI patients with an incomplete injury [2,68,69].

### 7.3. Epidural or Noninvasive (Transcutaneous) Electrical Stimulation of Spinal Cord

This is yet in its explorative phase. Studies have indicated a positive effect on NBD, but further research is called for [2,70,71,72].

### 7.4. More Neuromodulations

Other neuromodulations that can be mentioned are tibial nerve stimulation, dorsal genital nerve stimulation, abdominal wall stimulation, functional magnetic stimulation, and perianal electrical stimulation. These modalities still have little structural scientific evidence and their effects and indications are therefore yet to be properly determined [2,73,74]. Nevertheless, a pilot study performed by Sanagapalli et al. in 2018 shows promising results for posterior tibial nerve stimulation in individuals with MS suffering from FI [75].

Although still calling for further confirming results, SARS and SNS have been studied and applied within the SCI population to a greater extent than within the MS population. Nonetheless, more research is needed in both groups to further state the effects at time of intervention [26].

## 8. Discussion

Neurogenic bowel dysfunction is a complex and common condition among patients with SCI and MS. It is highly burdensome due to constipation, fecal incontinence, abdominal bloating, and pain. It is therefore of great importance that the condition is recognized early and that treatment is individualized to each patient [2,19,76]. Within the SCI population, it is estimated that up to 80% of patients struggle with constipation and 75% experience FI. Within the MS population, different data are presented, but it is usually estimated that minimum of two-thirds suffer from bowel symptoms [16,19,28].

Despite the high prevalence of NBD, structured research and evidence regarding NBD treatment are very limited. Most results report empirical recommendations rather than strong evidence-based results [77]. TAI is the modality with the most scientific evidence. Since various laxatives are yet to be compared in RCTs within the NBD population, recommendations regarding laxatives rely on research within the general population and idiopathic constipation or FI. This carries many limitations as the underlying pathophysiology is different. Moreover, constipation and FI are often present simultaneously within patients suffering from NBD, being a particular challenge of the condition. Results from studies on idiopathic conditions are therefore not necessarily applicable to the NBD population [16]. Moreover, surgical interventions have not yet been properly compared to one another.

Research regarding NBD within patients with MS is likewise scant. Recommendations are usually based on results from patients within the SCI group or idiopathic bowel symptoms, leaving clinicians with the above-mentioned limitations and the lack of a formalized approach regarding bowel symptoms [1,2,16,19,26,28,53].

The aim of the present paper is to shed light on the challenges of NBD and the current strategy of treatment and ultimately to present an updated, simplified treatment algorithm more coherent with current clinical practice. Current clinical practice usually follows a “treatment pyramid”, introducing the above-mentioned steps gradually, as demonstrated in Figure 1 [28]. This treatment pyramid, or versions thereof, is endorsed by many authors [2,19,26]. This stepwise approach is primarily based on empirical evidence and expert opinions [19]. Furthermore, the current pyramid suggests that all steps be tried out, including various experimental and surgical treatments. This is problematic in several ways. First, many of the experimental treatments as well as surgical interventions are highly specialized procedures (e.g., SARS and MACE) and therefore of limited availability. Hence, they cannot be offered at all treatment units around the world [2,67]. Second, not all patients are suitable candidates for many of these experimental and/or specialized treatments. These steps are not mandatory steps within the current clinical approach and therefore redundant as a part of the pyramid. Third, it would appear from the existing treatment pyramid that a colostomy is considered after a MACE has failed. This does not reflect the actual clinical approach, seeing as the different surgical interventions will not necessarily be deemed fit for all patients. Lastly, surgical procedures as several individual steps cause confusion, suggesting all modalities should be tried out, which is not necessarily clinically accurate.

It should be emphasized that surgery as a merged step is not to suggest that different surgical procedures are interchangeable. It is merely an attempt to avoid general confusion as to the need to perform all surgical and experimental possibilities before a stoma might become an option. We advocate that the different surgical possibilities, and when to introduce such intervention, be left to the patient and the treating surgeon specialized within the area. Surgery presented as a united and last fundamental step after TAI is therefore closer to clinical reality. We therefore present an updated version of the treatment pyramid (Figure 2), aiming to facilitate an approach towards NBD treatment and guide most clinicians. When to advance in the pyramid, add, or change treatment is yet to be determined. Therefore, as previously accentuated, an individual and continuous assessment is of essence and will depend on patient preference and local clinical resources. In this process, the MENTOR tool may assist clinicians in determining indications for change in bowel management or treatment.

In a recent review performed by Kurze et al., 2022, the authors call upon proper guidelines for NBD treatment within patients with spinal cord injury and/or disease due to an alleged lack thereof [78]. Their aim is to provide guidelines for clinicians, relying on the existing and above-mentioned treatment pyramid for NBD. However, NBD due to MS is not mentioned and, as stated in the current paper, it is analogous to NBD due to SCI and a highly extensive and disabling problem amongst MS patients. Furthermore, Kurze et al. do not revise the treatment pyramid itself, which the authors of the present paper believe is warranted and thus our purpose.

Although this paper centers upon NBD due to SCI and MS, it is worth mentioning that bowel symptoms are prevalent in several other neurological disorders as well. Examples are spina bifida, Parkinson’s disease, and stroke. However, many of these are of different character, although resulting in many of the same symptoms. After a stroke, constipation as well as dysphagia might become a problem; however, it depends on the location of the lesion. Likewise, NBD is a common complication of spina bifida. The pathophysiology can resemble that of SCI patients, although spina bifida not seldom entails concomitant hydrocephalus and, in some, intellectual deficits [22]. In PD, pathophysiology is fundamentally different from that of NBD and comprises a complex interplay of many factors, involving a lack of the neurotransmitter dopamine in both the brainstem and the enteric nervous system (ENS), autonomic neuropathy, and dystonia of the striated external anal sphincter muscle [79,80]. Hence, treatment of NBD in the above-mentioned conditions might require a somewhat different approach than in MS and SCI patients.

## 9. Conclusions

Neurogenic bowel dysfunction is very common in patients with SCI or MS. Symptoms of NBD include constipation, fecal incontinence, and abdominal discomfort. Treatment is challenging and structural research is sparse. The existing established treatment algorithm or “treatment pyramid” does not reflect the current clinical approach. Therefore, we propose a simplified version of the treatment algorithm to facilitate NBD treatment within the SCI and MS populations.

## Data Availability

Data are contained within the article.
